# Experimental study on the influence of longitudinal slope on airflow-dust migration behavior after tunnel blasting

**DOI:** 10.1038/s41598-023-46242-5

**Published:** 2023-11-13

**Authors:** Hui Hu, Yuchun Tao, Heng Zhang, Yiqi Zhao, Youdi Lan, Zhenghui Ge

**Affiliations:** 1 Key Laboratory of Transportation Tunnel Engineering, Ministry of Education, School of Civil Engineering, Southwest Jiaotong University, Chengdu, 610031 Sichuan China; 2 Department of Road and Bridge Engineering, Sichuan Vocational and Technical College of Communications, Chengdu, 611130 China; 3 Sichuan Highway Planning, Survey, Design and Research Institute Ltd, Chengdu, 610041 Sichuan China

**Keywords:** Civil engineering, Environmental sciences

## Abstract

In this paper, a 1:21 model experiment was conducted to discuss the dust diffusion efficiency and liner trolley obstruction effect inside the tunnel at − 9% to 9%, the effect of different initial dust concentrations on dust diffusion and liner trolley obstruction effect at 6% slope, and the effect of different return air velocity on dust diffusion at 6% slope, the reliability of the results is verified by computational fluid dynamics simulations. The results show that as the slope of the tunnel changes from 0 to − 9%, the average dust diffusion time decreases by 3.7% at the working face and the dust concentration difference between the front and rear of the trolley is improved by 2.7%. When the slope of the tunnel changes from 0 to − 9%, the average dust diffusion time increases by 7.2% at the working face and the dust concentration difference between the front and rear of the trolley is improved by 17.9%. With each 100 mg/m^3^ increase in the initial dust concentration, the dust diffusion time at the working face and the tunnel exit increases by 9.15% and 8.17% on average, and the lining trolley obstruction time increases by 23.33 s on average. The dust diffusion times take an average reduction rate of 15.7%, with the increase of return air velocity. The recommended return air velocity is greater than 1 m/s for large slope tunnels. When the slope changes from 0° to 9°, the hindrance rate of slope on dust diffusion is 2.88462%, 8.65385%, and 16.34615% respectively. Dust diffusion efficiency will be reduced as the tunnel slope changes from 0° to 9°, The growth rate of slope on dust diffusion is − 0.96154%, − 2.88462%, and − 6.73077% respectively.

## Introduction

With the effective depth of the “Western Development” policy, the continuous promotion of infrastructure construction in the western region of China, and high-speed railroad construction in western China in an orderly manner^1,2^, China’s western terrain is dominated by mountains, plateaus, and basins, with large topographic relief^3^. In the technical background of the traditional drill and blast method as the main boring process, large slope tunnels and spiral tunnels are mostly used to solve the problem of large driving detour distances and slopes between high-altitude areas and low-altitude areas^4–6^. According to the current specification, the slope of the tunnel main tunnel is generally bounded by 3%^7^, while in recent years some tunnel cross passage and inclined shaft projects have extremely large slope cases such as − 14° and 38° ^7,^^8,9^. When in large slope tunnel and tunnel construction, the buoyancy and gravity of the smoke will no longer be perpendicular to the ground, which makes the buoyancy and gravity become an additional driving force for the smoke to spread in the tunnel, which has an important impact on the pollutant concentration distribution. Construction ventilation, as the main way of air purification in underground space operations, is one of the key indispensable technical aspects of tunnel construction^10,11^. At the same time, if the exhaust gas, toxic gas, dust, and other pollutants generated from drilling, blasting, slurry spraying, slagging, and other operational processes cannot be discharged from the tunnel in time, it will seriously threaten the physical and mental health and life safety of the tunnel workers^12,13^.

At present, the research on the construction ventilation of long tunnels has achieved considerable results. These studies mainly focus on dust diffusion characteristics, temperature variation characteristics, optimization of ventilation parameters^14,15^. Some of these scholars have mainly used numerical simulations and field tests to study slope tunnel construction ventilation. Kong et al.^16^ used ANSYS to establish the ventilation model for tunnels with different slope changes, the flow field characteristics near the jet fan and the effect of tunnel slope on the ventilation efficiency were studied; Zhang et al.^17^ simulated the smoke diffusion effect of the shaft under the influence of longitudinal ventilation and slope to study the change in the rate of smoke emission from the shaft in the tunnel; Li et al.^18^ carried out a field test in an underground sloping tunnel and the results showed that the temperature decreases in the form of exponent; Rafael et al.^19^ learned influence of the slope in the ventilation semi-transversal system of an urban tunnel; Song^20^ studied the influence of slope and radius of curvature of the spiral tunnel on the diffusion pattern of pollutants.

At the same time, many scholars have also carried out a lot of research in the field of dust dispersion during underground construction through modeling tests, numerical simulations, field testing, orthogonal tests and so on. Su et al.^21^ investigated the wind-driven natural ventilation and dust suppression effects of coal sheds equipped with windbreak gables that have a porous structure. The investigation is conducted using a combination of wind tunnel experiments and computational fluid dynamics (CFD) numerical simulation; Guo^22^ used the virtual point source method, established the theoretical distribution model of the curved intersection tunnel to explore the distribution of dust that can be inhaled during the construction of drilling holes; Hou et al.^23^ developed an air-dust coupling model using the Euler–Lagrange method to study the dust migration law under various air conditions and the dust control mechanism in the large eddy, to address the challenging issue of dust diffusion pollution in tunnel excavation; Liu^24^ built an in-depth analysis of the effects of an air curtain generator’s radial and axial pressure airflow rate and a dust removal fan’s exhaust airflow rate on the diffusion and pollution behaviors of dust. In response to a large amount of dust generated during excavation in a large tunneling project, Ralf Irmler and Julian Buchner^25^ monitored whether certain geotechnical parameters of the rock could affect the quality and quantity of the dust, and the results showed that there was a correlation between water content and the quality and quantity of the dust; Liu^26^ used the numerical simulation method combined with field measurements to study the dust pollution diffusion law in a tunnel under different air suction volumes; Guo^27^ used the Ohl-Lagran method to simulate the combined motion of air and dust, and studied the time-spatial evolution of dust and inhalable particles that exploded under ventilation within 100 s of the explosion.

However, few studies are carried out on the construction ventilation of large slope tunnels, and few studies on the effect of large slope on the change of diffusion of pollutants distribution using model experiments. Therefore, because of the complex construction environment of sloped tunnels, an in-depth study of the spatial and temporal distribution of pollutants during the construction period, the investigation of their transport characteristics and derivative development mechanisms under the coupling effect of wind and flow fields, and the formulation of the effect of slope on the transport efficiency of pollutants to achieve efficient and safe construction with green construction as the core concept are the key issues that need to be solved in tunnel construction today.

In this paper, a 1:21 model experiment was conducted to discuss the dust diffusion efficiency and liner trolley obstruction effect inside the tunnel at − 9° to 9°, the effect of different initial dust concentrations on dust diffusion and liner trolley obstruction effect at 6° slope and the effect of different longitudinal return air velocities on dust diffusion at 6° slope. The results were validated by CFD numerical simulation.

## Model building and working condition setting

### Construction area WIND FLOW control equation and similarity theory

Generally speaking, the airflow in the tunnel is a complex three-dimensional turbulent flow. Still, the length of most tunnels is much larger than the hydraulic diameter of the tunnel. It can be considered that the airflow parameters approximately evenly distribute on the tunnel section, that is, the parameters such as airflow pressure, velocity, and density regard as constants in the same section. The following continuity equations and governing equations are used to calculate the airflow.

1 $$\frac{\partial \rho }{{\partial t}} + \frac{\partial }{{\partial x_{j} }}(\rho u_{j} ) = 0$$

2 $$\frac{\partial \rho }{{\partial t}} + \frac{\partial }{{\partial x_{j} }}(\rho u_{j} u_{i} ) = - \frac{\partial \rho }{{\partial x_{j} }} + \frac{\partial }{{\partial x_{j} }}\left[ {\mu_{e} \left( {\frac{{\partial u_{i} }}{{\partial x_{j} }} + \frac{{\partial u_{i} }}{{\partial x_{i} }}} \right)} \right] + S_{i}$$

where *ρ* Is the air density, *t* is the time, *u*_*J*_ is the air velocity in *X*_*J*_ direction, *μ* is the dynamic viscosity coefficient, *μ*_*e*_ is the equivalent dynamic viscosity coefficient, *S*_*i*_ is the generalized source term of momentum conservation equation; *κ* is the turbulent motion energy, *ε* is the energy dissipation rate of turbulent flow.

Similarity experiments are based on the principle of similarity, according to a certain scale to make a model with a similar scale to the prototype for experimental studies, in order to predict the flow phenomenon that will occur in the prototype, the core of the modeled similarity experiments is to reproduce the physical nature of the phenomenon of motion. In the case of experimental conditions, the model experiment is more effective instead of theoretical calculations^28,29^.

The gas–solid two-phase flow analysis is used to describe the movement of dust particles in the airflow in the tunnel, and the length, time and amount of material (force) are often taken as the basic quantities in the mechanical analysis, which are [*L*], [*M*] and [*T*], respectively. There are 7 main similarity parameters can be derived according to similarity criterion and basic equations of flow dynamics^9,10^:

3 $$S{\text{tk}} = \frac{{\delta^{2} \rho \lambda }}{{lv^{2} }}\quad \left( {{\text{Stokes}}\;{\text{number}}} \right)$$

where *Stk* is expressed as the ratio of the inertial force of dust particles to the resistance of the airflow, *δ* is the dust particle size, *ρ* is dust particle density, *λ* is the relative velocity of gas–solid two-phase flow, *l* is characteristic length(e.g. hydraulic diameter), *v* is fluid velocity;

4 $$S{\text{t}} = \frac{l}{vt}\quad \left( {{\text{Strouhal}}\;{\text{number}}} \right)$$

where *St* is a similarity criterion for characterizing flow nonstationarity, t is time;

5 $$E{\text{u}} = \frac{p}{{\rho_{1} v^{2} }}\quad \left( {{\text{Euler}}\;{\text{Number}}} \right)$$

where *Eu* is the characteristic number describing the momentum transfer, *p* is the pressure difference; *ρ*_*1*_ is the volumetric massl;

6 $$F{\text{r}} = \frac{{v^{2} }}{gl}\quad \left( {{\text{Froude}}\;{\text{number}}} \right)$$

where *Fr* is the characteristic number describing the relative magnitude of fluid inertia force and gravity force, *g* is the acceleration of gravity;

7 $$M{\text{a}} = \frac{v}{a}\quad \left( {{\text{Mach}}\;{\text{Number}}} \right)$$

where *Ma* is the characteristic number describing the degree of compressibility of the fluid, *a* is the speed of sound;

8 $$\Pr = \frac{{\mu c_{p} }}{k}\quad \left( {{\text{Prandtl}}\;{\text{Number}}} \right)$$

where *Pr* is the characteristic number describing the interaction of energy and momentum transport processes in a fluid, *μ* is dynamic viscosity coefficient, *k* is the coefficient of thermal conductivity, *c*_*p*_ is the isobaric specific heat capacity;

9 $${\text{Re}} = \frac{l\rho v}{\mu }\quad \left( {{\text{Reynolds}}\;{\text{number}}} \right)$$

where *Re* is a characteristic number describing the flow of a fluid.

In practical terms, attaining entire equality among comparable quasi-numbers is both unfeasible and unattainable, leading to inevitable conflicts between similar principles. Given that each similarity criterion is associated with a distinct physical interpretation, and these interpretations reflect different emphases among the criteria, it is possible to disregard certain subordinate criteria that have minimal impact on the phenomenon by examining the experimental requirements and the physical meanings of each criterion. Given the low Mach number of the airflow velocity in this particular model, the ambient temperature being constant, and the interior airflow exhibiting a consistent flow throughout lengthy ventilation, the parameters Ma (Mach number), St (Strouhal number), and Pr (Prandtl number) are not taken into consideration^30,31^.

**Figure 1:**
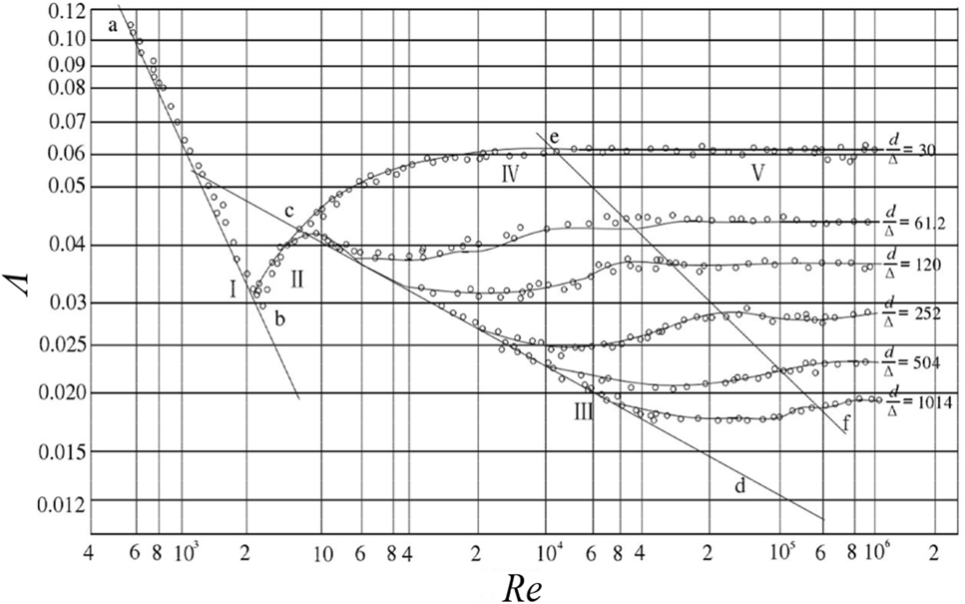
Nicholas curve.

To ascertain the most appropriate similarity criterion, it is imperative to establish the flow state of the airflow within the tunnel. Consequently, the Nicholac curve is employed for the purpose of quantification. Nikolausz conducted artificial rough tube flow studies to establish the correlation between the along-track drag coefficient and the Reynolds number and relative roughness. The experimental data was partitioned into five distinct regions, including the laminar flow region, crucial transition region, turbulent smooth region, turbulent transition region, and turbulent rough region. Figure 1 depicts the Nikolausz curve, illustrating the relationship between the along-track drag coefficient (*Λ*) and the Reynolds number (*Re*).

The experimental curves for the six tube flows in the figure have six different horizontal lines, indicating that *Λ* in the turbulent roughness region is related to *Δ/d* rather than *Re*. In the modeling experiments, if both the prototype and the model are in the drag square region ($Re > 4160\left( {\frac{d}{2\Delta }} \right)^{0.85}$), then only geometrical similarity (*Δ/d* being equal) is needed, not Re, to automatically achieve drag similarity. That is, by designing the model according to the Froude criterion, the viscous force similarity can be automatically realized without the need to satisfy the Reynolds similarity criterion.

Based on the above theory, the present prototype tunnel section is calculated. The calculation object includes the airflow state in the tunnel:

10 $$R{\text{e}}_{t} = 4160\left( {\frac{{d_{t} }}{{2\Delta_{t} }}} \right)^{0.85} = {170179}{\text{.13}} < {\text{Re}}_{{{\text{t0}}}} = \frac{{\rho_{a} v_{t} D_{t} }}{{\mu_{a} }} = {2}81716.16$$

where *Re*_*t*_ is the range of the square area of resistance to wind flow in the tunnel, *d*_*t*_ is the hydraulic diameter of the tunnel, *∆*_*t*_ is the absolute roughness of the tunnel wall, in accordance with the general situation of smoothing the selection of 0.1 m, *Re*_*t0*_ is the reynolds number of airflow inside the tunnel

Therefore, the Reynolds number of the airflow inside the tunnel is larger than the minimum value of the resistance square region, the flow state has entered the resistance square region, similar to the model in the Reynolds number criterion can be disregarded.

The Stokes criterion Stk, which is expressed as the ratio of the inertial force of dust particles to the resistance of the airflow, must be satisfied for dust diffusion tests. When the modeled dust reynolds number *Re* are in the same region as the prototype dust *Re*. The Euler number characterizes the action of surface forces and is the ratio of pressure to inertial forces, and two flows with equal Euler numbers indicate similar pressures. The Froude number characterizes the ratio of inertial force to gravity. Two flow response Froude number is equal, it means gravity is similar. Although the tunnel ventilation WIND FLOW by gravity is often negligible, but the blasting of soot settlement and diffusion by gravity is more significant. And when the flow of the main forces for viscous force, gravity and pressure, because the pressure is usually a strain, as long as the viscous force, gravity is similar, the pressure will be similar on its own, that is to say: when the Reynolds number, the Froude number is equal, then the Euler number by itself is equal. Therefore the similarity of the tunnel model follows the Stokes number and Froude’s criterion.

### Experimental model building

After determining the flow state of the airflow inside the tunnel and the selected similarity criterion, it is necessary to determine the composition of the model and the similarity ratio. The model similarity ratio *Π* is usually determined according to the requirements and scope of the test, taking into account the experimental site, modeling and measurement conditions.

**Figure 2:**
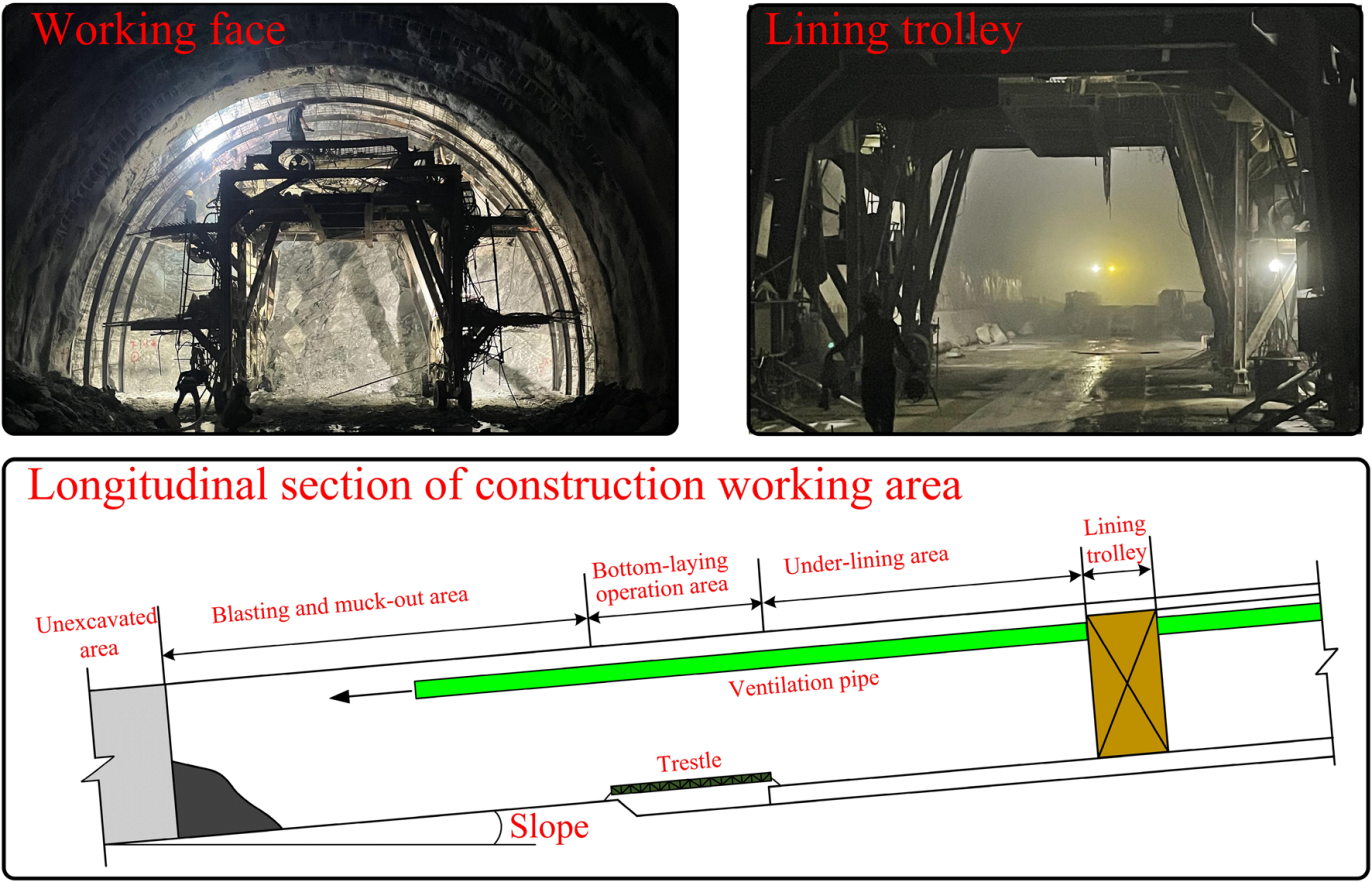
Railway tunnel working area.

The operational area for tunnel construction utilizing the drill-and-blast method primarily encompasses the span between the working face and the lining cart, which is illustrated in Fig. 2. Given that this particular domain serves as the primary focal point for tunnel construction personnel, with stringent requirements for environmental quality, we have chosen to investigate the operational segment of a substantial railway tunnel as the subject of our study. To facilitate our examination of the key parameters influencing dust diffusion, we have constructed a model of comparable scale.

The tunnel model refers to the standard section of a double-line railroad tunnel, and the scaled-down model is constructed within 250 m of the construction section, with a proposed prototype section area of 150 m^2^. According to the size of the experimental site, the test method and the tunnel prototype, the length similarity ratio *Π*_*l*_ is selected as 1:21.

**Figure 3:**
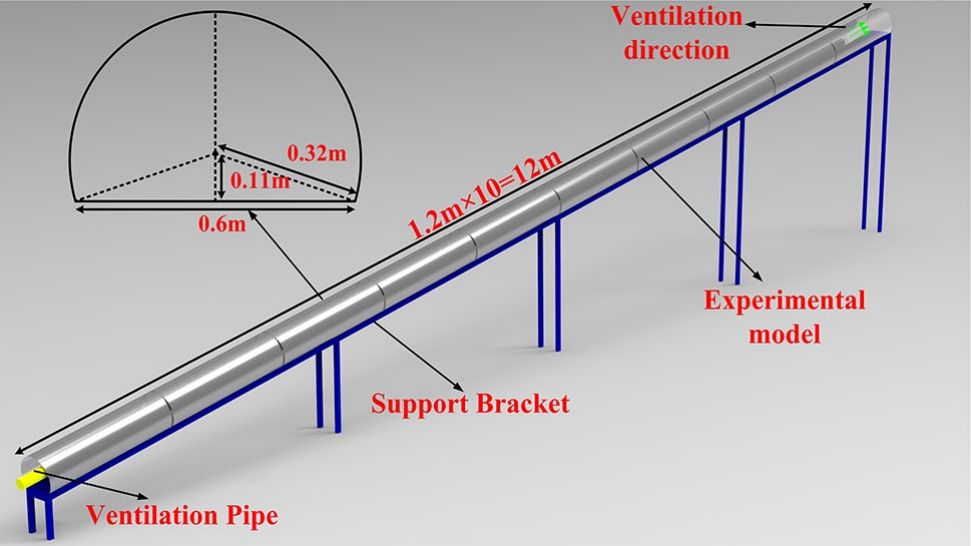
Schematic diagram of the model.

To achieve better observation effect, the experimental model is made of 2 mm thick transparent acrylic plate custom splicing, the length of single section model is 1.2 m, a total of 10 sections, the total length of the model is 12 m, using a 0.5 m high platform to support the placement. The specific dimensions of the section are shown in Fig. 3.

**Figure 4:**
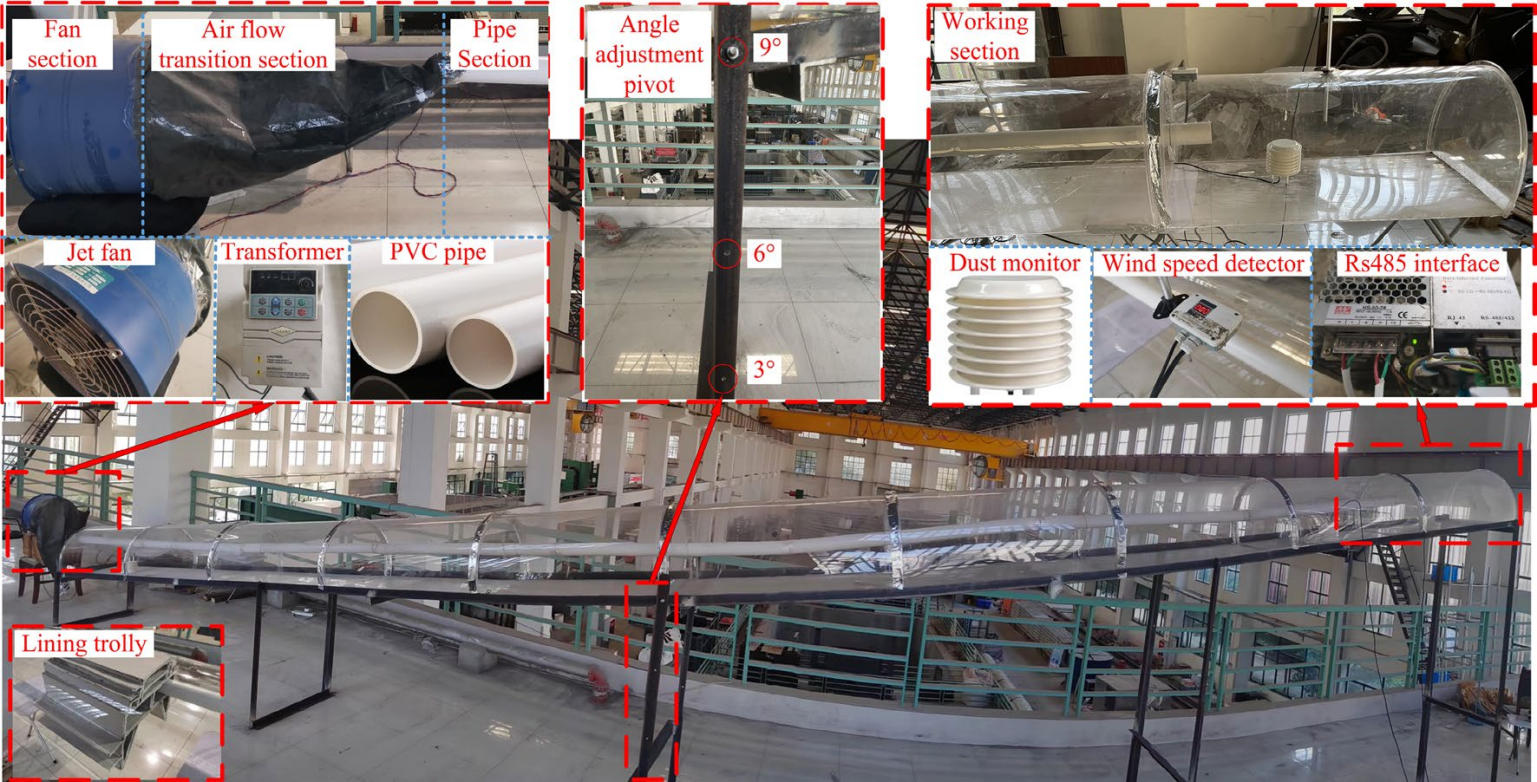
Tunnel model physical picture.

In order to maintain comprehensive airtightness throughout the experiment, the perimeters of each individual section of the model were effectively sealed using aluminum foil tape. The schematic diagram illustrating the construction of the model is presented in Fig. 4. In order to enhance the fidelity of the internal environment representation within the tunnel section, it is advisable to incorporate a rudimentary lining trolley model. This model should be constructed using 1 mm rigid cardboard material, measuring 0.47 m in length, and positioned at a distance of 9.5 m from the working face of the tunnel model (equivalent to a distance of 200 m from the corresponding prototype).

The ventilation system of the model experiment adopts a small axial fan of SF4-4R type, meanwhile, in order to ensure the ventilation effect, on the basis of not exploring the air leakage of the pipe and its form on the overall impact of the experiment, it is proposed to use 1 mm thick PVC rigid pipe as the WIND FLOW path of the whole model experiment (i.e. the prototype pipe), with 1800 mm diameter pipe as the prototype to build the model, with an inner diameter of 85 mm, and the air supply system through canvas At the same time, SZ-J/0.75G-A2 fan velocity control switch is used to control the axial fan velocity to obtain the predetermined return air velocity in the tunnel with 0.75 kW power; the air pipe outlet distance from the palm surface is 0.95 m (20 m for the prototype), and the model ventilation system is shown in Fig. 4.

According to the Nikolaus experiment, *Λ* is only related to *Δ/d* but not to Re in the square region of turbulent resistance. Therefore, in the modeling experiment, if both the prototype and the model are in the drag square region, then the drag similarity (*Λ* is equal) can be achieved as long as the geometrical similarity is satisfied without the need for *Re* to be equal. In other words, by designing the model according to the Froude criterion, viscous force similarity can be achieved automatically without having to satisfy the Reynolds number similarity criterion at the same time. Therefore, the ventilation resistance similarity needs to pay attention to the absolute roughness of the material to satisfy the similarity ratio, and the calculated need to control the roughness is about 0.009 m, while the acrylic plate roughness is generally controlled in the 1 μm or less, which meets the demand.

From the previous section, it is known that the Reynolds number *Re*_*t*0_ > 2 × 10^5^ in the original tunnel, the Reynolds number Re_m_ of the model for the check:

11 $$Re_{m} = \frac{{\rho_{a} v_{m} D_{m} }}{{\mu_{a} }} = {1354167}{\text{.02}} > {200000}$$

In essence, the hydrodynamic perspective establishes a similarity rule between the Reynolds number of the experiment and the Froude number in the flow field between the experimental and actual tunnels. In a similar vein, the Stokes number provides evidence supporting the validity of the similarity rule in elucidating the behavior of dust particles in both scenarios.

### Experimental dust setting

The applicability of the similarity criterion for dust particle size is limited to dust flows consisting of particles of a single size. However, it should be noted that the dust particle size distribution in the actual project differs from this ideal scenario. In situations where the particle concentration is very low, such that particle collisions are not readily apparent, it is possible to simplify the representation of multiple-sized particles by employing a single characteristic size. This characteristic size can be determined as the biggest particle size within the system. In situations where the concentration of particles is relatively low, such that there is minimal occurrence of particle collisions, it is possible to simplify the representation of multi-sized particles by using a single characteristic size. In this case, the characteristic size can be determined as the biggest particle size.

In the construction of the drill and blast method, drilling, blasting, slag transportation, shotcrete, and other construction processes will generate a large amount of dust, according to the source is divided into the original dust, blasting dust and process dust, blasting dust and process dust is 80% to 90% of the total dust production. The dust generated by blasting is mainly the dust formed by the rock being broken, accompanied by the dust caused by the huge blast force; the source of dust from slag transportation is mainly the transfer of slag and dust from operating machinery; the dust generated by shotcrete is mainly generated by the diffusion of concrete powder, the collision between high-speed concrete and tunnel walls and mutual collision. Tunnel blasting dust particle size is generally 0–75 μm^32,33^.

**Figure 5:**
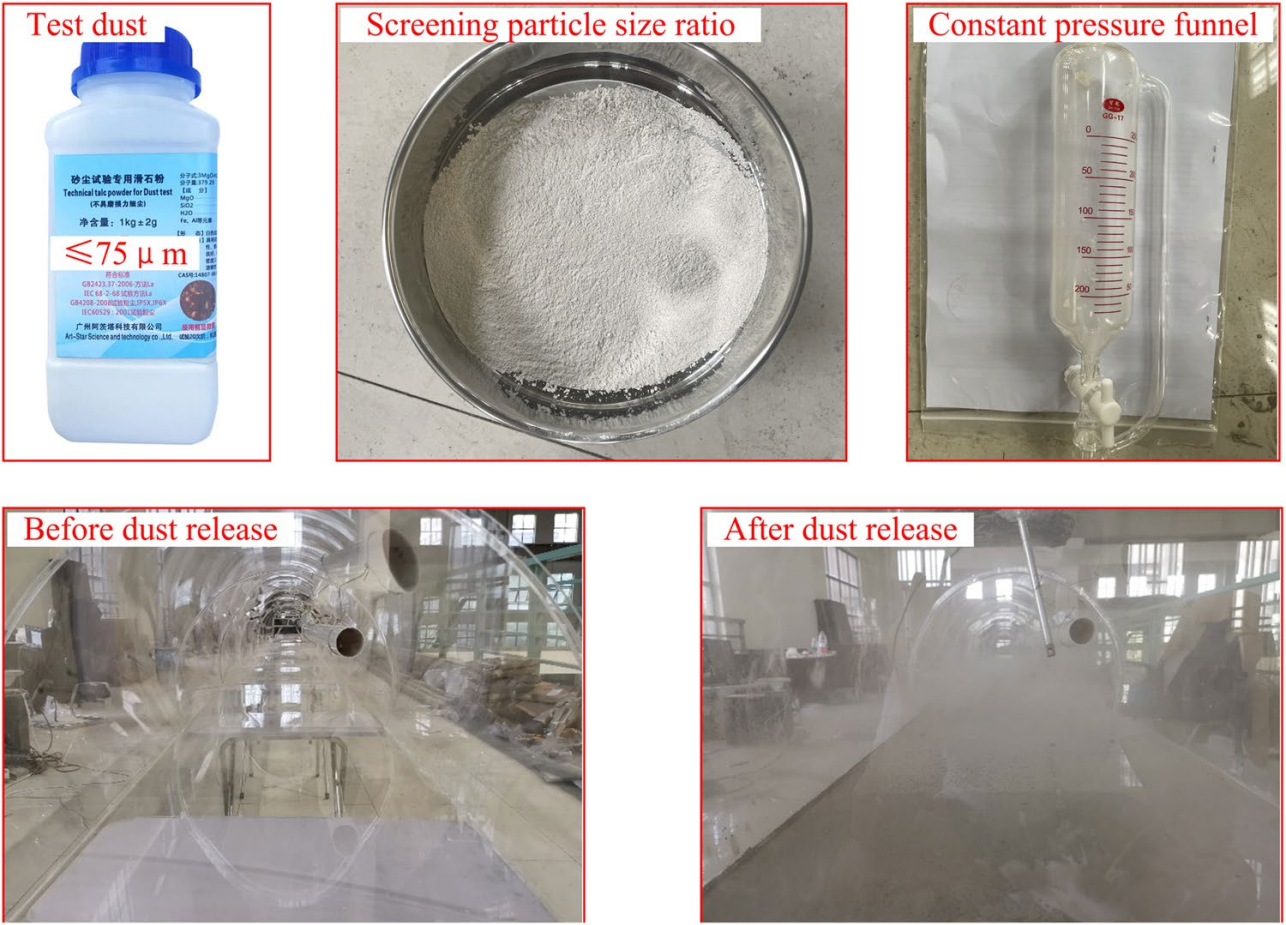
Experimental dust disposal.

The standard port 50 ml constant pressure funnel with tetrafluorine piston valve is used as a dust generator to circumvent the disturbance of its initial distribution field morphological characteristics during the large-scale non-uniform intake of dust particles and restore the original state of blasting floating dust after stagnation as much as possible. To simulate the initial dust concentration field of different scales during the experiment, the sieved dust was introduced into the constant pressure funnel, the piston was opened, and the dust particles entered the model tunnel at a uniform velocity and in equal amounts. The ventilation dust concentration monitoring device is placed at the front of the tunnel model, and when the concentration near the palm surface reaches the predetermined experimental working condition, the ventilation system is turned on and the experimental monitoring is carried out. Experimental dust disposal progress and equipment are shown in Fig. 5.

### Monitoring setting

**Figure 6:**
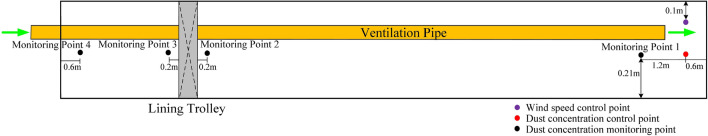
The layout of measurement points.

In order to examine the distribution pattern of dust under the influence of coupling effects, the experiment employs return air velocity and dust as the primary monitoring parameters. Real-time monitoring is conducted on the return air velocity and dust volume concentration. The XZ4451D-500MT-05088 type hot film return air velocity sensor is utilized to measure the return air velocity within a range of 0–5 m/s, with a precision of 3%, thereby determining the velocity in the specified section. The experimental apparatus is depicted in Fig. 6. The ventilation of the 1200 s was observed under various conditions, and data were collected at intervals of 10 s for monitoring purposes. The laboratory in which the experiment was conducted maintained an ambient temperature ranging from 19 to 22 °C, while the atmospheric pressure was measured at 101 kPa.

### Working condition setting

The experiment intends to explore the key factors affecting the dust migration characteristics during the tunnel construction period, taking the dust particle size, initial concentration, return air velocity, and second lining step as the main research objects, and measuring the spatial and temporal distribution of dust at different particle sizes, different sizes of initial dust concentration fields, different return air rates and different second lining steps. The monitoring contents and working conditions are listed in Table 1 below, with 13 working conditions in total.

**Table 1:** Working conditions setting.

Conditions	Consideration	Slope (%)	Air velocity (m/s)	Dust concentration (mg/m^3^)
1–1	Slope	0	1	200
1–2		3		
1–3		− 3		
1–4		6		
1–5		− 6		
1–6		9		
1–7		− 9		
2–1	Return air velocity	6	0.2	200
2–2			0.5	
2–3			1	
2–4			2	
3–1	Initial dust concentration	6	1	100
3–2				200
3–3				300
3–4				400

According to current specifications and engineering examples, the slope of tunnels, auxiliary passages, and inclined shafts generally does not exceed 3%, and there are few cases of large slopes. In this study, to investigate the extreme cases, we set the slope of ± 3%, ± 6%, ± 9%, and 0% as the tunnel slope variables, where positive and negative represent the downhill and reverse slope cases, with 7 control groups. Also, for the field measurement results of relevant long-drawn railroad tunnels, subject to various unfavorable factors in the actual ventilation system deployment and implementation, and the cross-sectional area is relatively large, the cross-sectional return return air velocity of long-drawn tunnels is generally less than 2 m/s and maintained at 0.4–0.8 m/s level. Therefore, 0.5 m/s, 0.8 m/s, 1.0 m/s, 1.5 m/s, and 2.0 m/s are taken as the return air velocity variables, and a total of 5 sets of working conditions are set. The dust concentration after tunnel working face blasting is determined by the construction environment, surrounding rock conditions, explosive type, dosage, etc. The initial dust concentration of 100 mg/m^3^, 200 mg/m^3^, 300 mg/m^3^, and 400 mg/m^3^ is used as the initial dust concentration variables in this study.

## Model test results discussion

### Impact of different slopes

**Figure 7:**
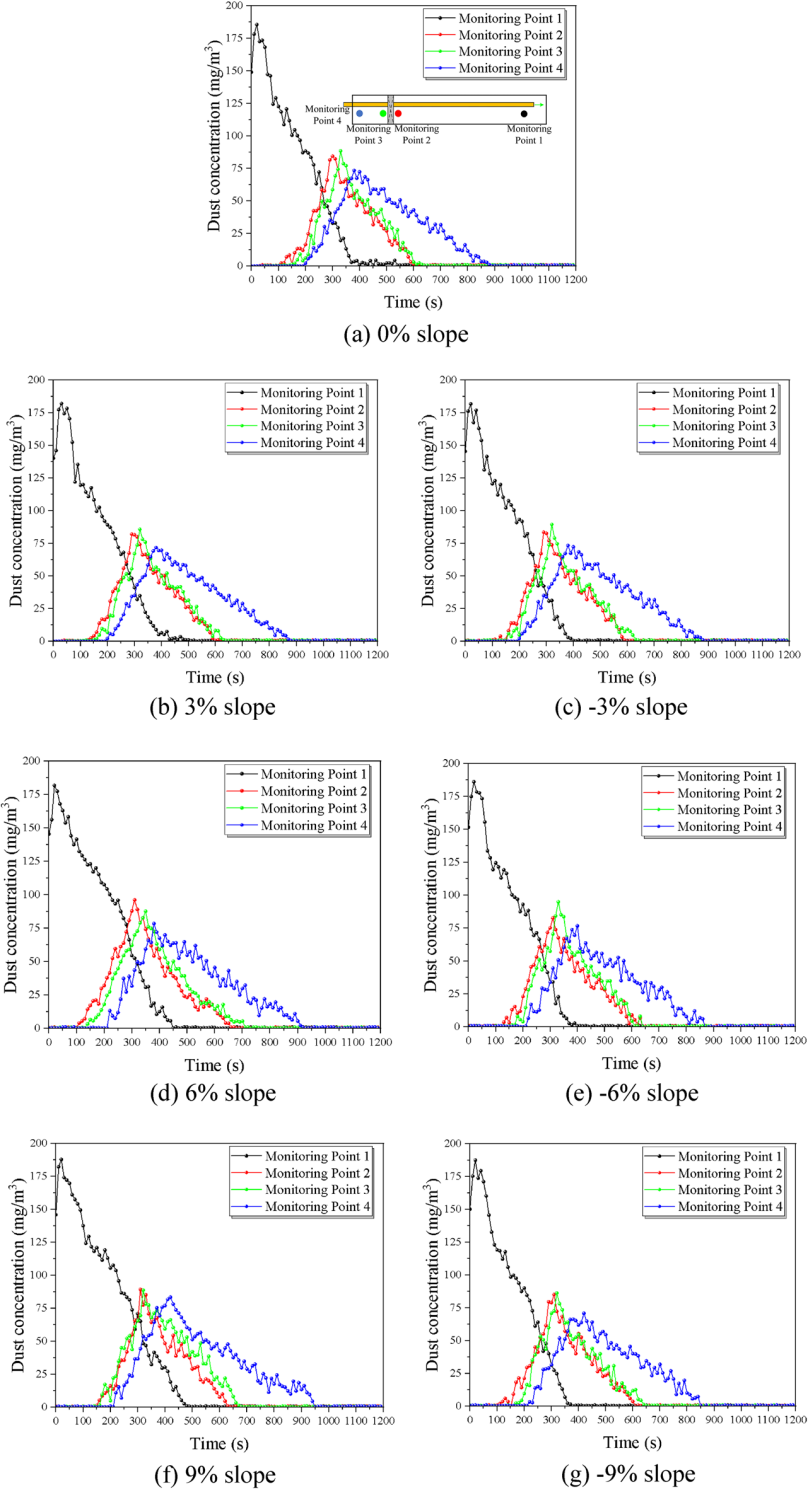
Comparison of dust discharge under different tunnel slopes.

To investigate the effect of different slopes on the dust diffusion law, the experiment was designed with seven groups of comparison conditions, and the tunnel slopes were 0%, ± 3%, ± 6%, and ± 9% for a total of seven different particle size ranges of dust for the control experiment, ensuring the same initial concentration of 200 mg/m^3^, controlling the model internal return air velocity of 1.0 m/s by adjusting the transformer. The variation of dust concentration with ventilation time at the location of the monitoring point was recorded and is shown in Fig. 7 below.

As shown in the Fig. 7, the dust concentration at the working face decreases rapidly after reaching the peak value, which is due to the fact that the large-size particles in the dust multiply and fall rapidly under the effect of gravity, and the small and medium-size particles diffuse towards the exit direction under the effect of wind flow. With the passage of time, the dust mass is continuously diluted by the wind flow in the tunnel and transported to the exit, so overall the further away from the working face of the monitoring point of the monitored dust peak value is lower, the longer the dust retention time.

Comparing the changes in dust concentration with time at each slope, it can be seen that the effect of different tunnel slopes on dust diffusion is mainly manifested by the increase of dust peak and the increase of time required for dust discharge with the increase of slope. Under various working conditions, the concentration of monitoring surface 1 has a small local rebound. The above situation is because as the slope of the model becomes larger, the dust is more likely to gather in the tunnel during ventilation, making it difficult for some of the dust to settle inside the tunnel or be discharged under the action of gravity. According to the comparison of the concentration data of the two supervisory surfaces at the front and rear ends of the trolley, the lining trolley has a significant hysteresis effect on dust diffusion. By processing the test data of the two monitoring points, the average dust concentration difference and dust diffusion lag time comparisons for each slope in Table 2 were obtained. From the table, it can be seen that the concentration difference from − 9 to 0% is slowly increasing with an average increase of 2.7%, dust diffusion lag time shows consistency, with reduced dust lag time at − 9% operating conditions. The concentration difference from 0 to 9% is significantly increasing with an average increase of 17.9%, and dust lag time improved by 20 s on average from 3 to 9%.

**Table 2:** Comparison of dust diffusion before and after lining trolley at different slopes.

Working conditions (%)	Average dust concentration difference (mg/m^3^)	Dust diffusion lag time (s)
− 9	6.01	20
− 6	6.19	30
− 3	6.438	30
0	6.508	30
3	7.753	30
6	8.95	50
9	10.65	70

**Figure 8:**
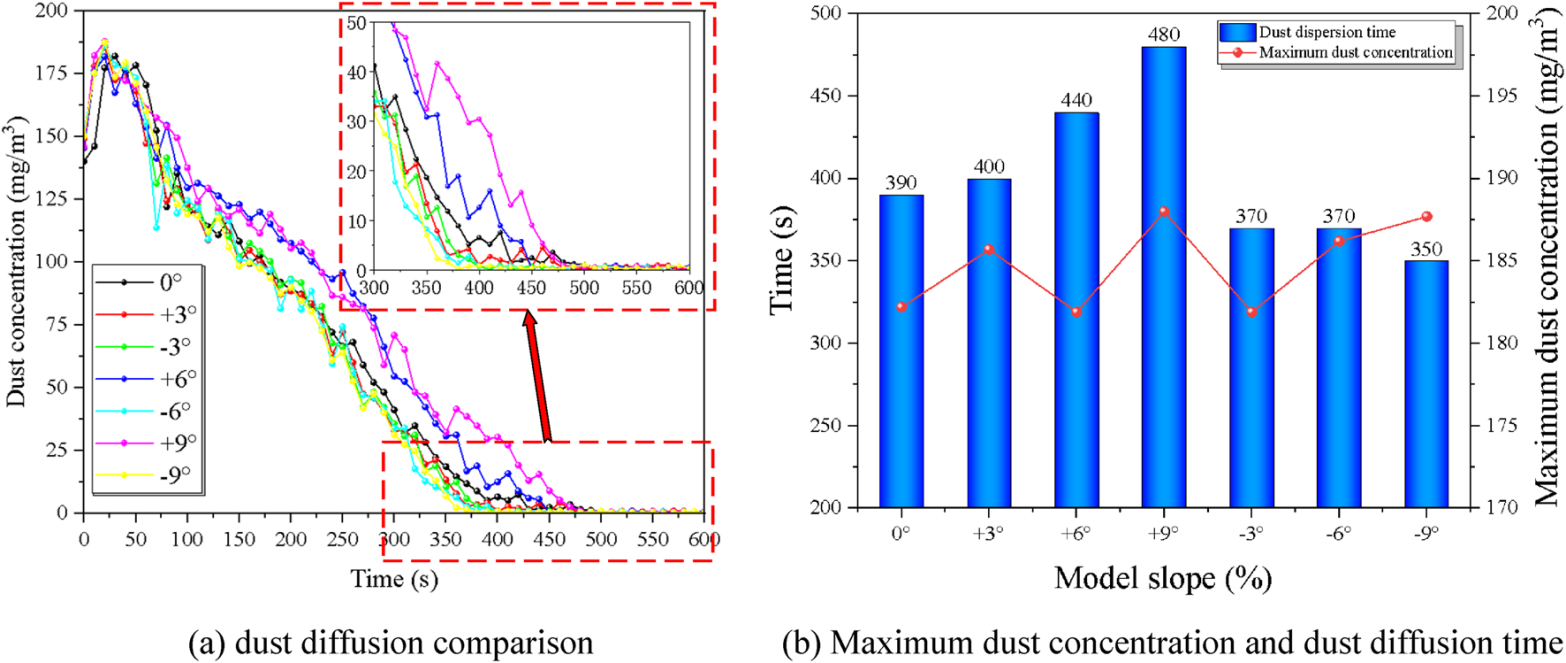
Comparison of dust diffusion by the slope at monitoring point 1.

From the Fig. 8, the maximum dust concentration is 187.7, 186.2, 181.9, 182.2, 185.7, 181.9, and 188.0 mg/m^3^ respectively, there is no major difference between the operating conditions. The dust concentration at each monitoring section near the working face gradually rises with time until the peak under the coupling effect of the wind flow field and then decreases with a decreasing trend of decreasing rate, and the monitoring section concentration at the working face under the slope of − 9%, − 6%, − 3%, 0%, 3%, 6%, and 9% working conditions reaches the specified threshold value with return air velocity increments of 350 s, 370 s, 370 s, 390 s, 400 s, 440 s, 480 s respectively. It can be found that from 0 to − 9%, the dust diffusion rate is partially increased, with a maximum difference of 50 s and an average growth rate of 3.7%; while from 0 to 9%, the dust diffusion rate is significantly reduced, with a maximum difference of 90 s. The average reduction rate is 7.2%.

### Impact of different initial dust concentration

**Figure 9:**
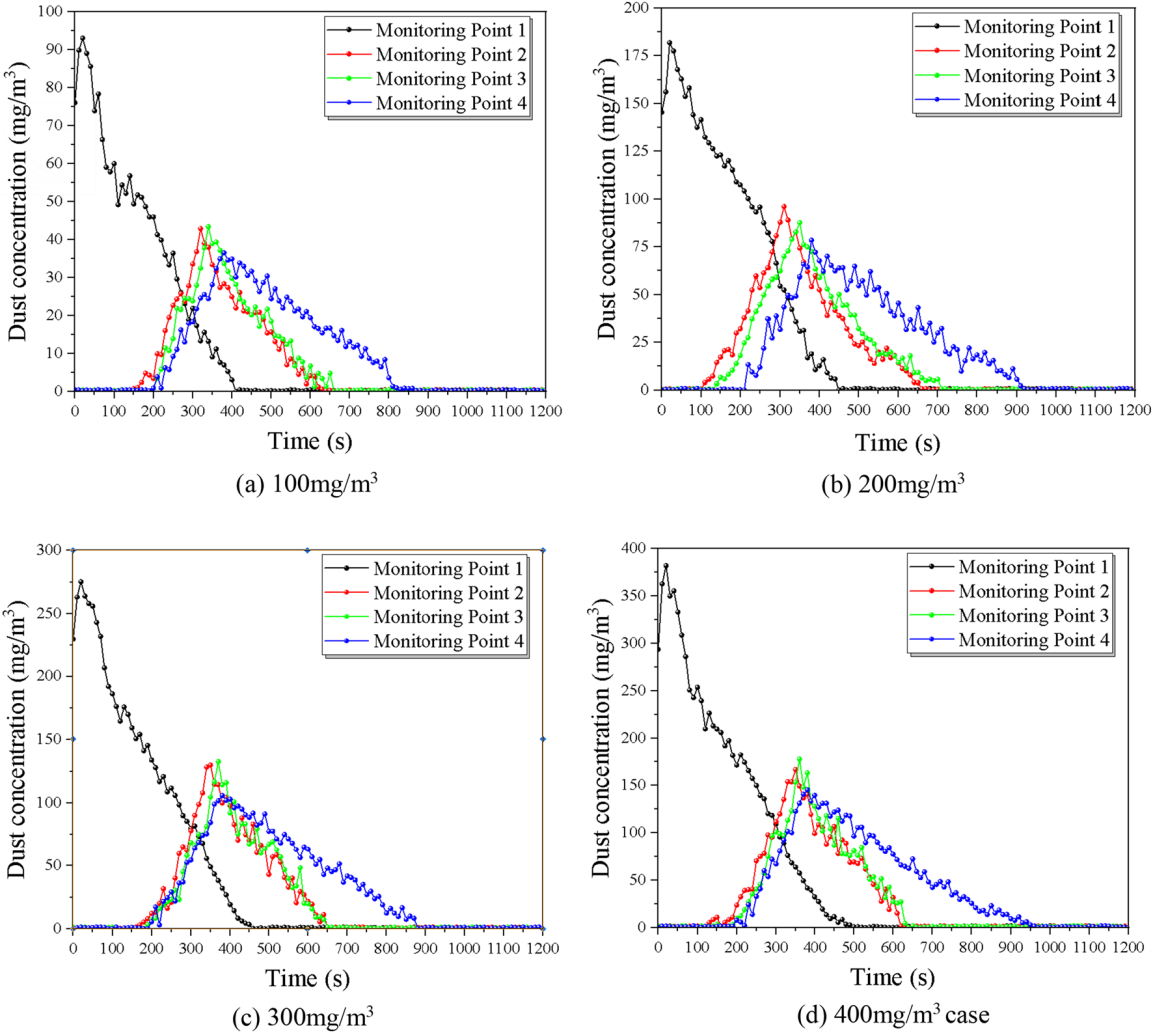
Comparison of dust discharge under different initial dust concentration.

To investigate the effect of different slopes on the dust diffusion law, the experiment was designed with seven groups of comparison conditions, and the tunnel slopes were 100 mg/m^3^, 200 mg/m^3^, 300 mg/m^3^, and 400 mg/m^3^, for a total of seven different particle size ranges of dust for the control experiment, controlling the tunnel slope as 6% by adjusting the support frame and return air velocity of 1 m/s by adjusting the transformer. The variation of dust concentration with ventilation time at the location of the monitoring point was recorded and is shown in Fig. 9 below.

The effect of initial dust concentration on dust diffusion mainly shows that the concentration peak rises with the increase of concentration field, the dust spreading rate decreases, and the lag degree of monitoring section position increases before and after the trolley, with each 100 mg/m^3^ increase in the initial dust concentration, the lining trolley obstruction time increases by 23.33 s on average. Under different initial dust concentrations, the overall change pattern of dust concentration with time gradient did not change significantly, and it showed a short time rise to the peak with the increase of time, and then the rate decreased. The initial dust concentration affects the peak concentration of the cross-section, which shows that the initial dust volume rises, the peak concentration rises, and the time of the peak concentration then lags, and the time used to reduce to the maximum allowable concentration lags accordingly. To further explore the effect of the initial dust volume on the dust diffusion efficiency of the tunnel, the following: monitoring point 1 and monitoring point 4 were selected to compare the variation of dust concentration in the internal section with time at different initial concentrations.

**Figure 10:**
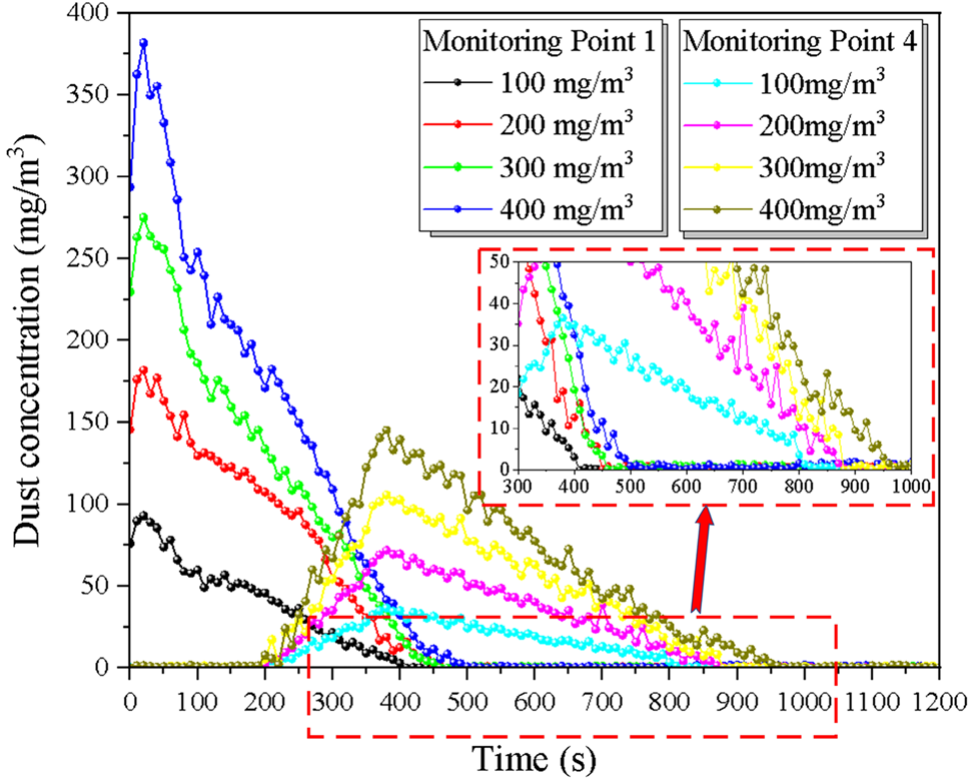
Comparison of dust diffusion by the slope at monitoring points 1 and 4.

It can be seen from the Fig. 10 that the time of maximum concentration of each condition is relatively consistent. As the initial dust concentration increases in monitoring point 1, there is a significant rebound in dust concentration after ventilation starts. The dust diffusion time increases significantly as the initial dust concentration increases, the dust diffusion time is 400 s, 440 s, 470 s, and 520 s respectively with an average growth rate of 9.15%. The dust arrival time at the outlet is not much different for each working condition, while the diffusion time also increases significantly with the increase of the initial dust concentration, the dust diffusion time is 590 s, 660 s, 690 s, and 750 s respectively with an average growth rate of 8.17%. This is mainly due to the increase in the initial dust concentration, the more stable the dust community, the relatively small effect of diffusion by the wind flow, requiring longer ventilation time to dilute and diffuse the dust.

### Impact of different wind velocity

To investigate the effect of different slopes on the dust diffusion law, the experiment was designed with seven groups of comparison conditions, and the return air velocity were 0.2 m/s, 0.5 m/s, 1 m/s, and 2 m/s, for a total of 4 different particle size ranges of dust for the control experiment, ensuring the same initial concentration of 200 mg/m^3^, controlling the tunnel slope as 6% by adjusting the support frame. The overall trend of dust concentration with time gradient in each monitoring section under the coupling effect of the wind flow field is: rising to the peak within a short time, and then gradually decreasing with time in a power function-like trend, i.e., the rate of decrease is first fast and then slow and gradually decreasing.

**Figure 11:**
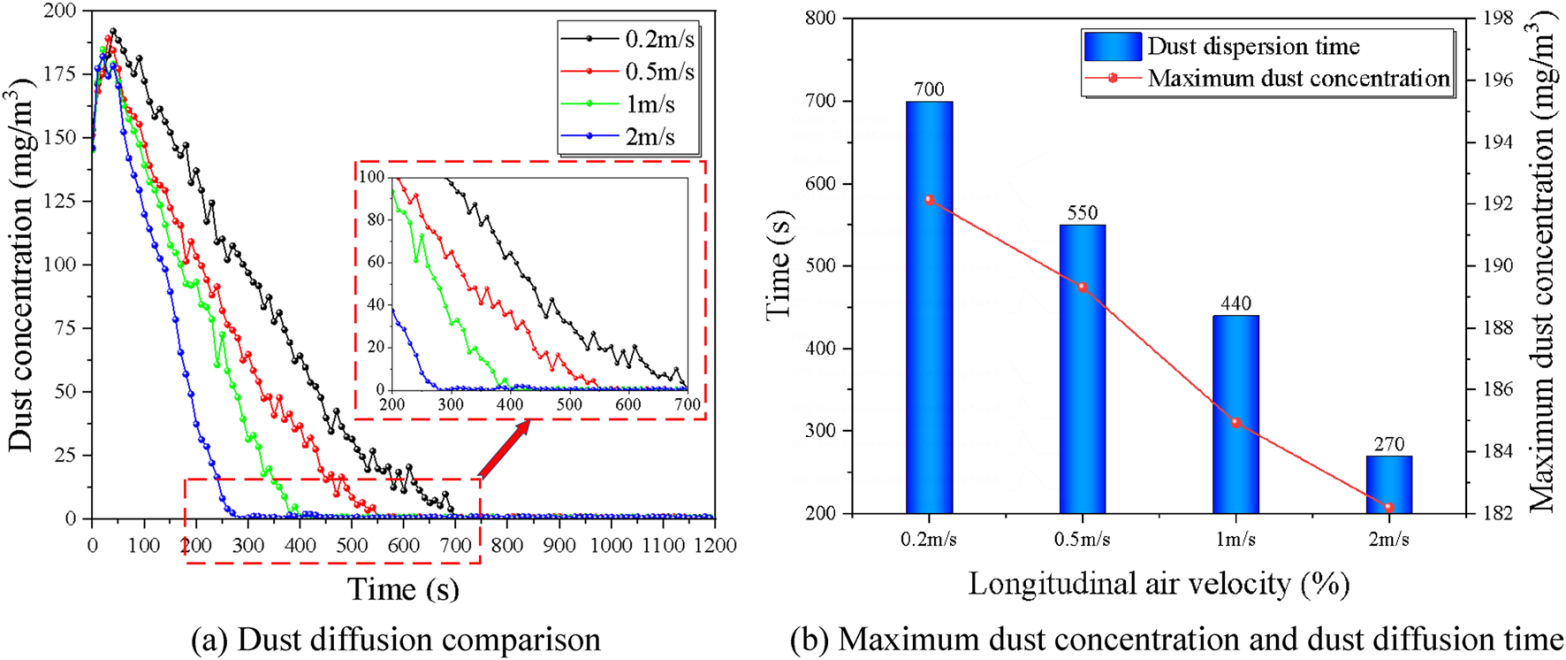
Comparison of dust diffusion by the slope at monitoring point 1.

**Figure 12:**
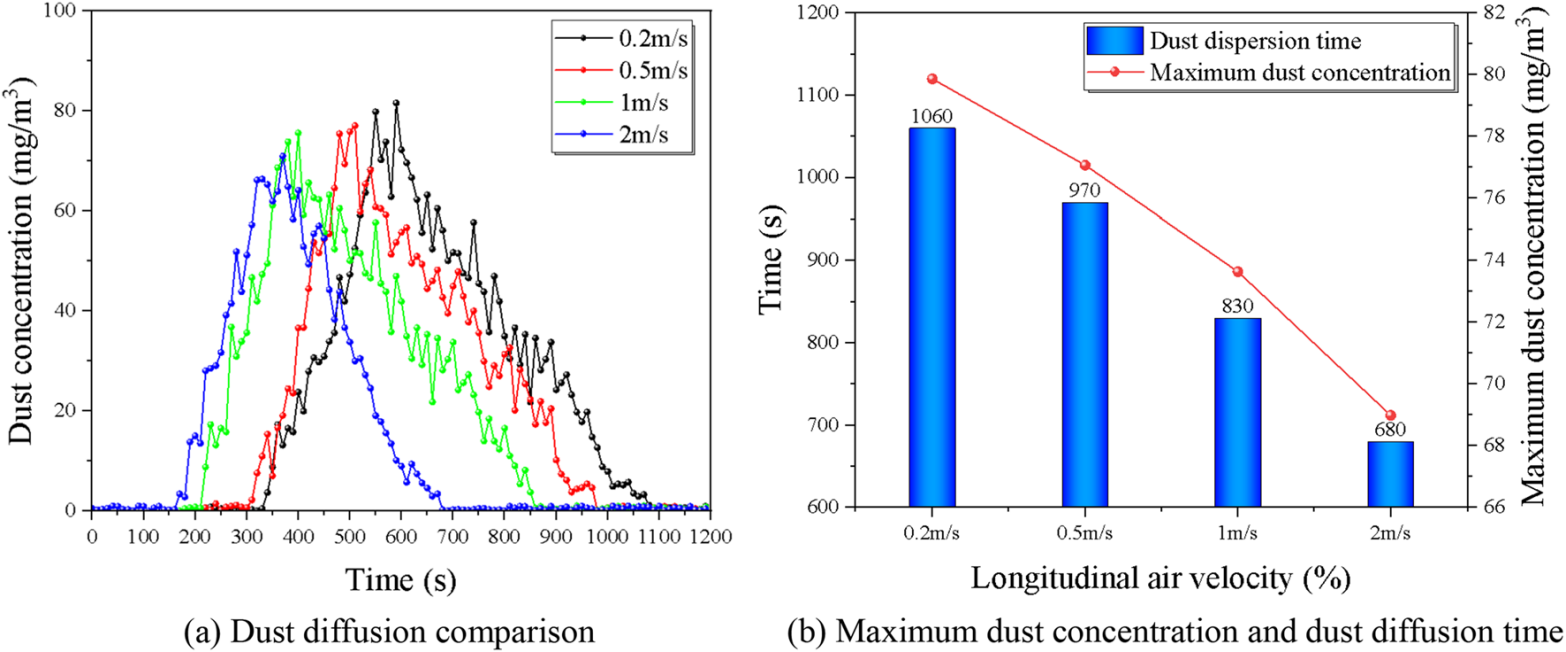
Comparison of dust diffusion by the slope at monitoring point 4.

The key characteristics of monitoring point 1 and monitoring point 4 are compared and analyzed under different return air velocity, see Fig. 11 and Fig. 12 below, it can be seen that the influence of different longitudinal wind return rates on its concentration changes is mainly manifested as the increase in return air velocity, the peak concentration of each monitoring section decreases, and the peak occurs earlier; at the same time, the reduction rate of the section concentration is enhanced to a certain extent. The peak dust concentration represents the overall dust pollution level in the tunnel, the time of peak appearance can represent the dust diffusion rate in the tunnel, and the time taken to reduce to the allowable concentration can reflect the dust removal efficiency under the existing ventilation system. The effects of return air velocity on the above three parameters are studied separately, mainly as follows.

Figure 12 shows the time of peak dust concentration at different return air velocitys near the working face, it can be seen that the time of peak concentration near the working face decreases with the increase of return air velocity, and the rate of decline increases with a certain weak trend, and the rising trend becomes more and more significant when the return air velocity is greater than 1.0 m/s. The average reduction rate was 26.7%, and the dust diffusion time was 700 s, 550 s, 440 s, and 270 s for the return air velocity rate of 0.2 m/s to 2 m/s, respectively, which showed a significant increase in dust removal effect.

**Figure 13:**
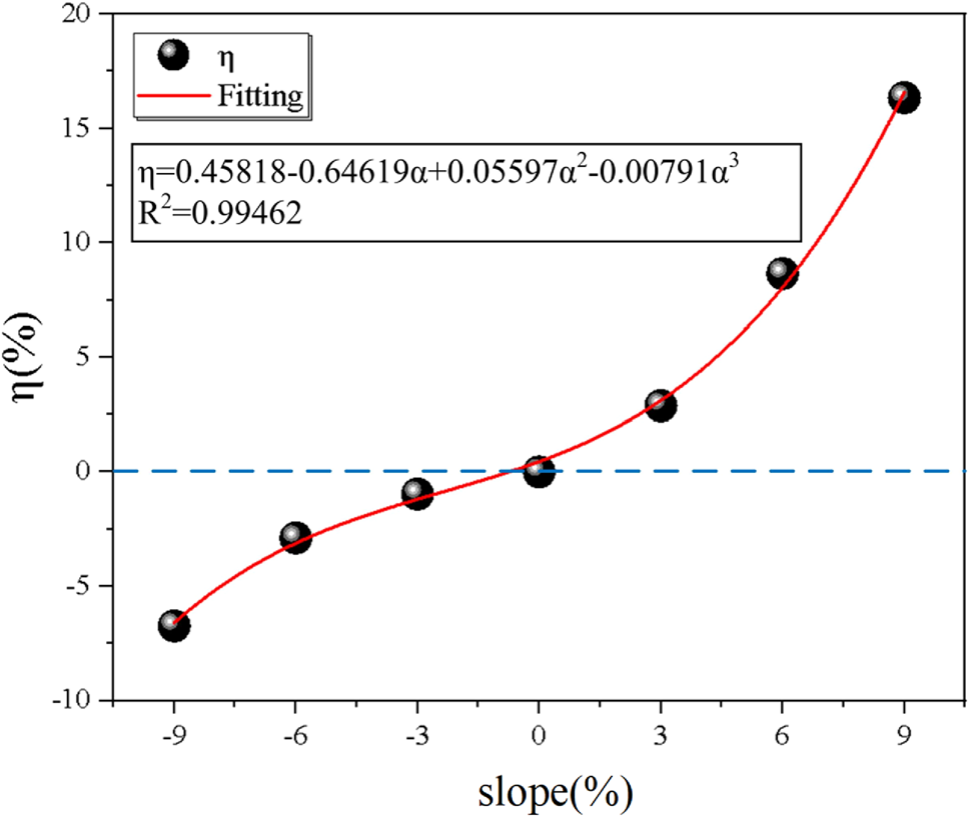
Dust diffusion influence factor of different slopes.

In Fig. 13, it can be found that the dust diffusion time and the peak concentration at the exit of the dust model are decreasing from 0.2 to 2 m/s. The dust diffusion times are the 1060 s, 970 s, 830 s, and 680 s, with an average reduction rate of 15.7%. The dust diffusion efficiency at 1 m/s and 2 m/s was significantly improved, with 14.4% and 24.7%, respectively. This indicates that the increase of the ventilation return air velocity can promote the transport of dust in the tunnel and accelerate the reduction of dust concentration, which means maintaining a longitudinal return return air velocity of more than 1 m/s in the tunnel under large slope conditions can significantly improve the efficiency of dust diffusion and improve the construction environment in the tunnel.

### Impact of dust diffusion influence factor

To specifically quantify the effect of tunnel slope on dust diffusion efficiency, select data for different slopes at monitoring point 4, and the dimensionless number η is defined to represent the dust diffusion influence factor. *η* is mainly expressed in the experiment as the length of dust discharge time and the time of maximum dust concentration appearance, also compares dust diffusion efficiency at 0%. When *η* is a positive number means that it hinders dust diffusion while a negative number means that it has a positive effect on dust diffusion. Therefore, in order to explore the relationship between the tunnel slope α and η, the relationship equation of *η* is defined as follows:

13 $$\eta_{i} = \frac{{t_{i} }}{{t_{0} }} \cdot \frac{{T_{i} }}{{T_{0} }} - 1$$

where* t*_x_ is the time of maximum dust concentration; *T*_x_ is the total time for dust diffusion in the lining trolley section.

The data from the exit monitoring points *t*_*i*_ and *T*_*i*_ at each slope are brought into Eq. (13) to obtain the data, and the curve is fitted using a polynomial, as shown in Fig. 12. The relationship equation between *η* and slope *α* is:

14 $$\eta = 0.45818 - 0.64619\alpha + 0.05597\alpha^{2} - 0.00791\alpha^{3}$$

where a is the slope of the tunnel; η is the total time for dust diffusion in the lining trolley section.

The fit coincidence *R*^*2*^ is 0.99462. It can be seen from the figure that compared to the no-slope case, the hindering effect of dust diffusion under the downhill longitudinal slope is greatly increased. The reverse slope situation has a negative effect on dust diffusion as the growth rate of *η* in 3%, 6%, and 9% are 2.88462%, 8.65385%, and 16.34615% respectively. The resistance to dust diffusion increases with the increase of tunnel slope, α shows a positive significance for dust diffusion efficiency, while the *η* in − 3%, − 6% and − 9% are − 0.96154%, − 2.88462%, and − 6.73077% respectively.

## Numerical simulation validation

### Basic assumptions of CFD numerical simulation

In order to verify the rationality of Eq. 4, this chapter will use CFD-Fluent software to establish a tunnel model of 1:1 to simulate the dust diffusion carried out by the model test. Computational Fluid Dynamics (CFD) is a branch of fluid dynamics, which is a cross-science combining modern fluid mechanics, numerical mathematics and computer science. It takes electronic computer as a tool, applies various discrete mathematical methods, and conducts numerical experiments, computer simulations and analytical researches on all kinds of problems in fluid mechanics to solve various practical problems.

For the complex and cumbersome practical problems to establish a mathematical model and solve the problem must be reasonably simplified, highlighting the main factors of the problem, ignoring the secondary issues, compression of the amount of calculations. In this regard, there are four assumptions that need to be made when modeling the gas–solid coupled flow dynamics in tunnels.

1. It is assumed that the airflow in the tunnel meets the ideal air state;
2. The air in the tunnel is assumed to be incompressible;
3. The WIND FLOW in the tunnel is assumed to be steady;
4. It is assumed that the WIND FLOW in the tunnel conforms to the continuity law.

### Geometric modeling and grid building

**Figure 14:**
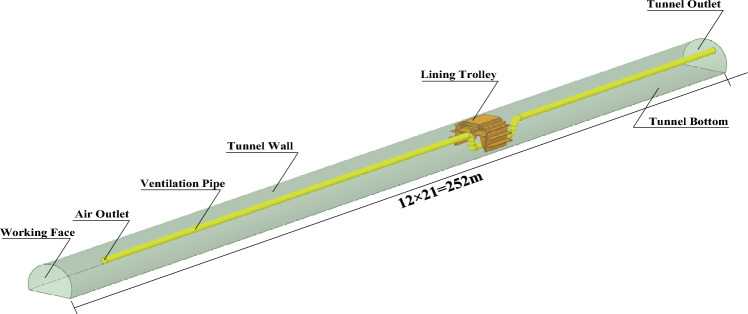
dust diffusion influence factor of different slopes.

In this study, the tunnel is modeled by SPACECLAIM 2022 software in 1:1 full-size. Calculated working conditions refer to the − 9% to 9% slope conditions in Table 1. Take the 0% gradient model as an example, the longitudinal length of the model is 252 m. The model contains the main body of the tunnel, ventilation ducts and lining carts, as shown in Fig. 14.

**Figure 15:**
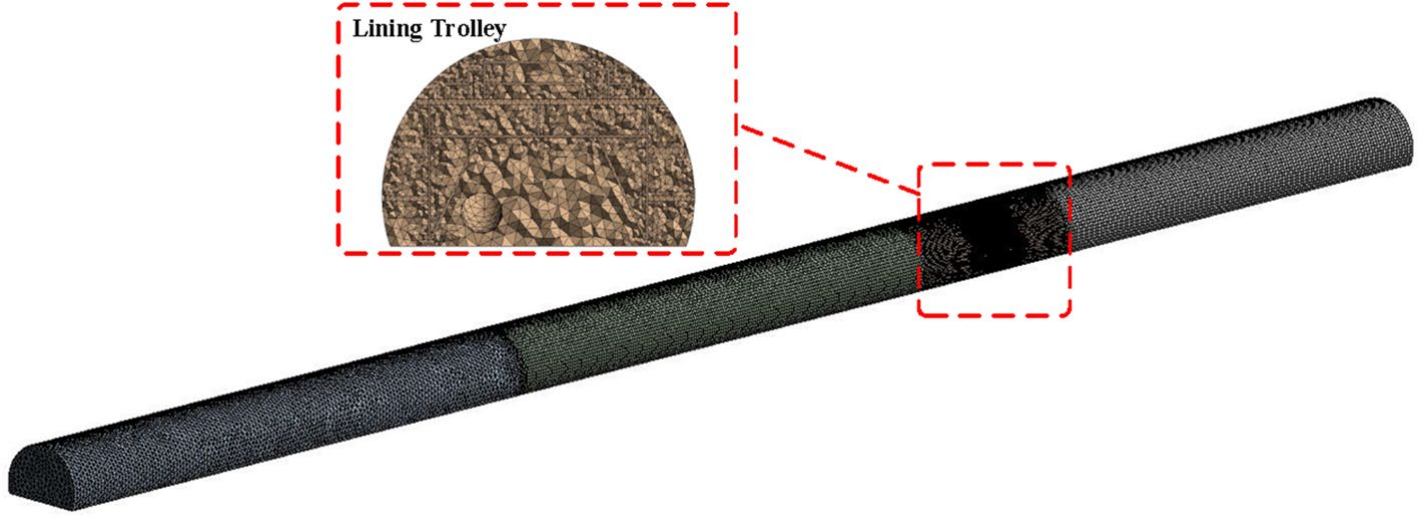
Model grid division.

HYPERMESH is used to mesh the 3D model, considering the relative complexity of the location of the tunnel’s connecting channels, in order to reduce the cost of calculations, a hybrid mesh is considered to be used, and the overall tunnel segments are structured hexahedral mesh, in order to ensure the accuracy of the wind flow simulation, the lining cart segments are encrypted for the local mesh. The grid size is 0.6, and the local encryption is 0.4 and the overall quality of the mesh is controlled to be over 0.8, with a total number of meshes of 426,595, and the mesh The total number of grids is 426,595, and the grid division is shown in Fig. 15.

### Boundary conditions and calculation model settings

Based on the previous similar proportion and model test research content, combined with FLUENT simulation and dust movement mathematical model, so as to determine the simulation of dust transport law simulation in the tunnel boundary conditions and simulation parameters.

According to the analysis of the tunnel environment in the previous section, the pressure-based transient solver is selected for calculation, and the SIMPLE algorithm is used to solve the coupled pressure equations. The turbulence model is the *k-ε* double equation model. The second-order implicit algorithm is chosen for the momentum, energy, turbulent kinetic energy, and eddy dissipation equations.

In order to simulate the work surface dust sprayed after blasting construction, the calculation opens DISCRETE PHASE MODEL (DPM), simulation of dust sprayed from the working surface, after the release of dust, turn off the injection source and start the fan to start the dust diffusion. The important relevant parameters are set as shown in Tables 3, 4:

**Table 3:** Working conditions setting.

Boundary	Define
Return air velocity	14.54 m/s
Air inlet boundary type	Velocity-inlet
Tunnel outlet boundary type	Outflow
Tunnel wall boundary type	Reflect
Tunnel wall roughness constant	0.01 m
Tunnel bottom boundary type	Trap

**Table 4:** Discrete phase model setting.

Discrete phase model	Define
Injection type	Surface
Diameter distribution	Rosin–Rammler
Min. diameter	0.1 μm
Max. diameter	75 μm
Mean. diameter	20 μm
Spread parameter	10 μm
Initial velocity	0 m/s

### Numerical simulation results and analysis

**Figure 16:**
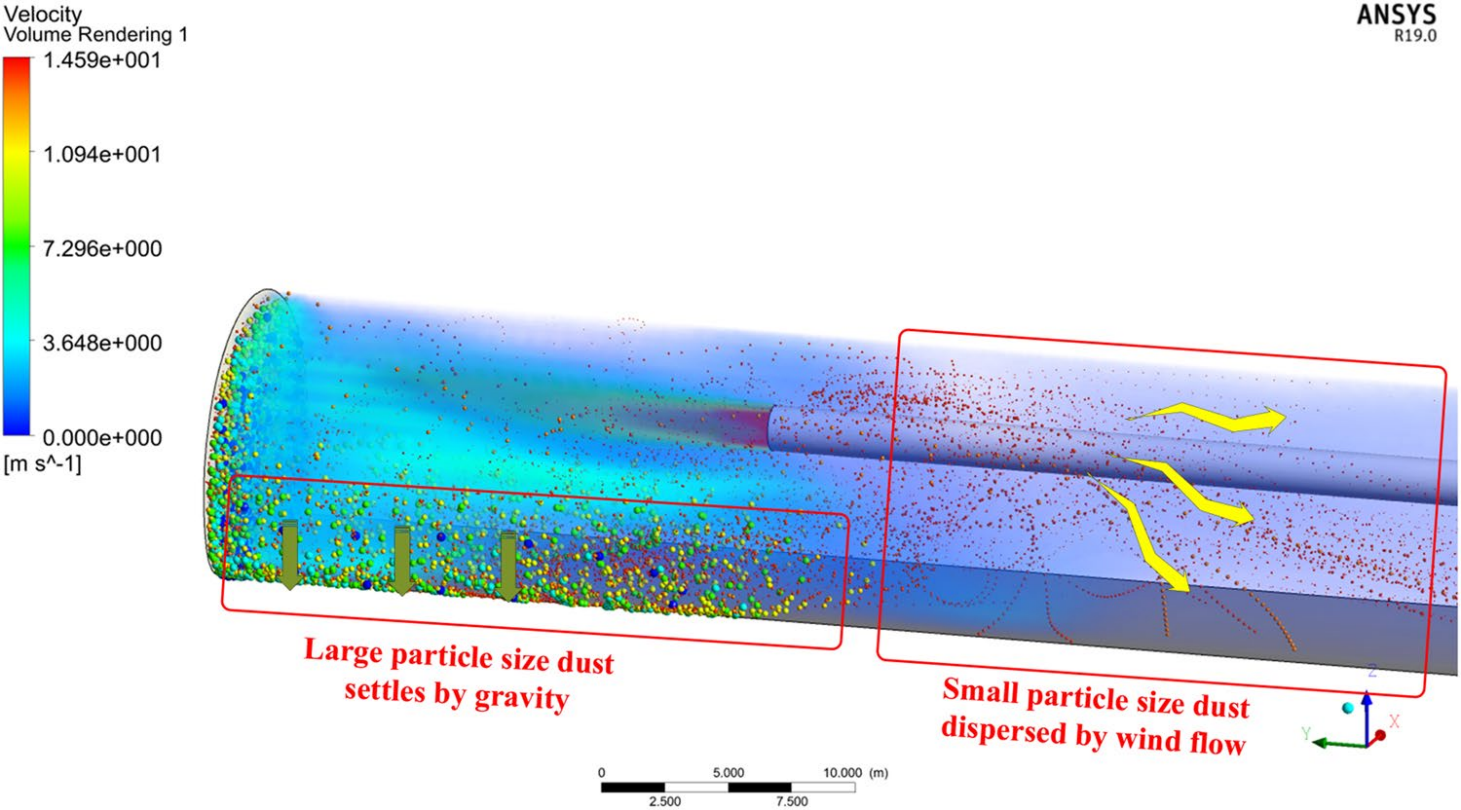
Dust diffusion at the working surface.

After the ventilation commenced, the computational cloud diagram of the working face is illustrated in Fig. 16. It can be seen that after 60 s of ventilation, some of the large mass dust particles begin to drop to the bottom and are caught at the bottom. Part of the large mass dust particles ejected through the walls and ducts did not settle to the ground immediately, and some of the particles began to diffuse out of the tunnel along with the wind flow inside the tunnel, and some of the medium-sized particles and some of the small-sized particles affected by the wind flow were captured at the bottom. Therefore, the movement pattern of dust in the numerical simulation findings is compatible with the expectation.

**Figure 17:**
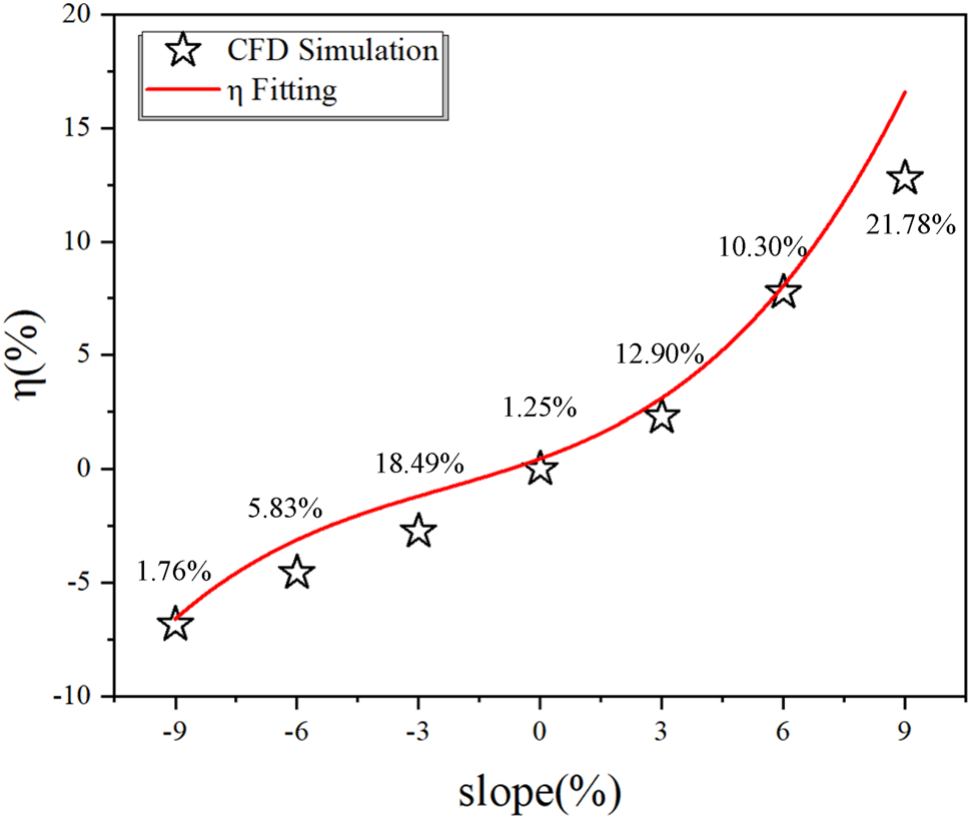
Comparison of numerical simulation results.

The results of the numerical simulation of each working condition are brought into Eq. (13) for calculation, and the simulation results are obtained, and compared with the results in the previous model test, and the following charts are obtained, with the percentage of error under each working condition labeled in the Fig. 17.

From the Figure, it can be seen that the numerical simulation results are in overall good agreement with the model test results. It is evident that the numerical simulation exhibits a satisfactory agreement with the model test outcomes, with an average discrepancy of 8.69%, when the slope is less than zero. Conversely, when the slope exceeds zero, the numerical simulation consistently yields lower results compared to the model test outcomes, particularly evident in the 9% condition where the error reaches 21.78%. Furthermore, the average error in this scenario amounts to 14.99%. The numerical simulation findings exhibit a tendency towards linear trends rather than curved patterns.

The discrepancy between the error of numerical simulation results and model test results is commonly attributed to the boundary conditions employed in the numerical simulation. Specifically, the boundary conditions in the simulation solely account for reflection from the wall, whereas in reality, the wall also captures a portion of the dust particles. Consequently, the numerical simulation results exhibit a greater number of dust particles being reflected by the wall and subsequently diffusing further within the tunnel, leading to a reduction in efficiency. The roughness of the wall in the model test will be augmented by the diffusion of dust particles, however the numerical simulation fails to accurately capture this phenomenon, resulting in a discrepancy between the simulated and actual outcomes.

## Conclusion

This paper reveals the understanding about the influence of longitudinal slope on airflow-dust migration behavior after tunnel blasting. A series of model tests with a scale of 1:21 are carried out to investigate the effect of the different slopes, initial dust concentration and return air velocity in the tunnel on the dust diffusion efficiency. The main results are as follows:

1. As the slope of the tunnel changes from 0 to − 9%, the dust diffusion efficiency in the tunnel improves. The average dust diffusion time decreases by 3.7% at the working face and the dust concentration difference between the front and rear of the trolley is improved by 2.7%. When the slope of the tunnel changes from 0 to 9%, dust diffusion in the tunnel is significantly lagging. The average dust diffusion time increases by 7.2% at the working face and the dust concentration difference between the front and rear of the trolley is improved by 17.9%.
2. When the tunnel slope is 6%, the initial dust concentration affects the time of the peak concentration then lags, and the time used to reduce to the maximum allowable concentration lags as the initial dust concentration increases. With each 100 mg/m^3^ increase in the initial dust concentration, the dust diffusion time at the working face and the tunnel exit increases by 9.15% and 8.17% on average, and the lining trolley obstruction time increases by 23.33 s on average.
3. When the tunnel slope is 6%, with the increase of return air velocity, the dust diffusion times take an average reduction rate of 15.7%. The dust diffusion efficiency is significantly improved while the return air velocity is 1 m/s and 2 m/s with the dust diffusion efficiency improved by 14.4% and 24.7% respectively. The recommended return air velocity is greater than 1 m/s for large slope tunnels.
4. The dust diffusion influence factor η represents the influence of the slope of the tunnel on the diffusion of dust to the exit. The results show that when the slope changes from 0 to 9%, the hindrance rate of slope on dust diffusion is 2.88462%, 8.65385%, and 16.34615% respectively. Dust diffusion efficiency will be reduced as the tunnel slope changes from 0 to 9%, The growth rate of slope on dust diffusion is − 0.96154%, − 2.88462%, and − 6.73077% respectively.
5. The Dust diffusion efficiency formula is verified by numerical simulation, and the two results are well fitted, when the slope is < 0%, the numerical simulation fits well with the model test results with an average error of 8.69%, and when the slope is > 0% the average error also reaches 14.99%, and the numerical simulation results are more inclined to a straight line rather than a curve.

## Data Availability

All data, models, and code generated or used during the study appear in the submitted article.
